# Finite element analysis part 1 of 2: Influence of short stem implant polyethylene configuration on glenohumeral joint biomechanics

**DOI:** 10.1002/jeo2.70000

**Published:** 2024-09-19

**Authors:** Geoffroy Nourissat, Victor Housset, Jean‐Marie Daudet, Léo Fradet, Rohan‐Jean Bianco, Uma Srikumaran

**Affiliations:** ^1^ Groupe Maussins Clinique Maussins Nollet‐Ramsay Santé Paris France; ^2^ Hôpital Henri Mondor Université Paris‐Est Créteil Créteil France; ^3^ FX Shoulder Solutions Viriat France; ^4^ Philomec Inc. Montréal Québec Canada; ^5^ Department of Orthopedic Surgery The Johns Hopkins University Baltimore Maryland USA

**Keywords:** anatomic total shoulder arthroplasty, bone stress, finite element model, polyethylene shape, reverse shoulder arthroplasty

## Abstract

**Purpose:**

Stress shielding in short‐stem arthroplasty can cause critical metaphyseal bone loss. If the size and shape of the humeral shaft are important factors, it is unknown whether the shape of the polyethylene component in reverse shoulder arthroplasty (RSA) affects bone stress around or within the stem. We explored the impact of polyethylene shape on humeral and scapular stress distribution using a finite element model.

**Methods:**

We developed a shoulder‐specific finite element model. A defined set of muscle forces was applied to simulate movements. An intact rotator cuff state and a superior deficient rotator cuff state were modelled. We used the FX V135 short stem in three conditions: total shoulder arthroplasty (TSA), and RSA with symmetrical and asymmetrical polyethylene (145°/135°). We measured biomechanical markers related to bone stress for different implant sizes. Joint kinematics and the mechanical behaviour of the implant were compared.

**Results:**

Rupture of the supraspinatus muscle produced a functionally limited shoulder. The placement of an anatomic TSA with an intact rotator cuff restored function similar to that of a healthy shoulder. RSA in the rotator cuff‐deficient shoulder restored function regardless of stem size and polyethylene shape. While stem size had an impact on the stress distribution in the bone and implant, it did not show significant potential for increasing or decreasing overall stress. For the same stem, stress distribution at the humerus is different between TSA and RSA. Polyethylene shape did not alter the transmission of stress to the bone in RSA. Asymmetric polyethylene produced a greater abduction range of motion.

**Conclusions:**

In terms of bone stress distribution, smaller stems seemed more appropriate for TSA, while larger stems may be more appropriate for RSA. Polyethylene shape resulted in different ranges of motion but did not influence bone stress.

**Level of Evidence:**

Diagnostic Tests or Criteria; Level IV.

**Abbreviations**
RSA: reverse shoulder arthroplasty
TSA: total shoulder arthroplasty

## BACKGROUND

Humeral stress shielding is a major concern in short stem anatomic total shoulder arthroplasty (TSA) and reverse shoulder arthroplasty (RSA) because it can cause critical metaphyseal bone loss and fractures around the stem [15, 17, 40]. Several elements may contribute to stress shielding, including the size and shape of the humeral shaft [16, 30], but it is unknown whether the shape of the polyethylene component (i.e., humeral insert) influences bone stress around or within the humeral stem. Polyethylene shape changes the position of the humeral head relative to the glenoid. This geometrical modification can have an impact on abduction initiation and maximal range of motion. This needs also to be quantified in parallel with bone and implant stress. Finite element models have been used to study the mechanisms of shoulder mobility and stability in both healthy and rotator cuff‐deficient configurations [2, 18, 45]. Concerning RSA, finite element studies have evaluated risk factors for augmented stresses at the interface of humeral components and the risk of bone resorption and stress shielding. Stress distribution is different between TSA and RSA [2, 18, 45]. No study has evaluated the difference of the stress shielding of the same short stem design in TSA and RSA configuration. Furthermore, to our knowledge, no study has evaluated the role of the shape of the polyethylene humeral liner and short stem size in RSA.

Our objective was to explore the impact of polyethylene component shape and stem size on postoperative shoulder biomechanics in short humeral stems with a focus on bone stress and to evaluate if the stress shielding around the stem is the same between anatomic and reverse configuration. Our hypothesis was that both of the tested parameters have different influences on postoperative glenohumeral biomechanics.

## MATERIALS AND METHODS

### Finite element model description

**Figure 1:**
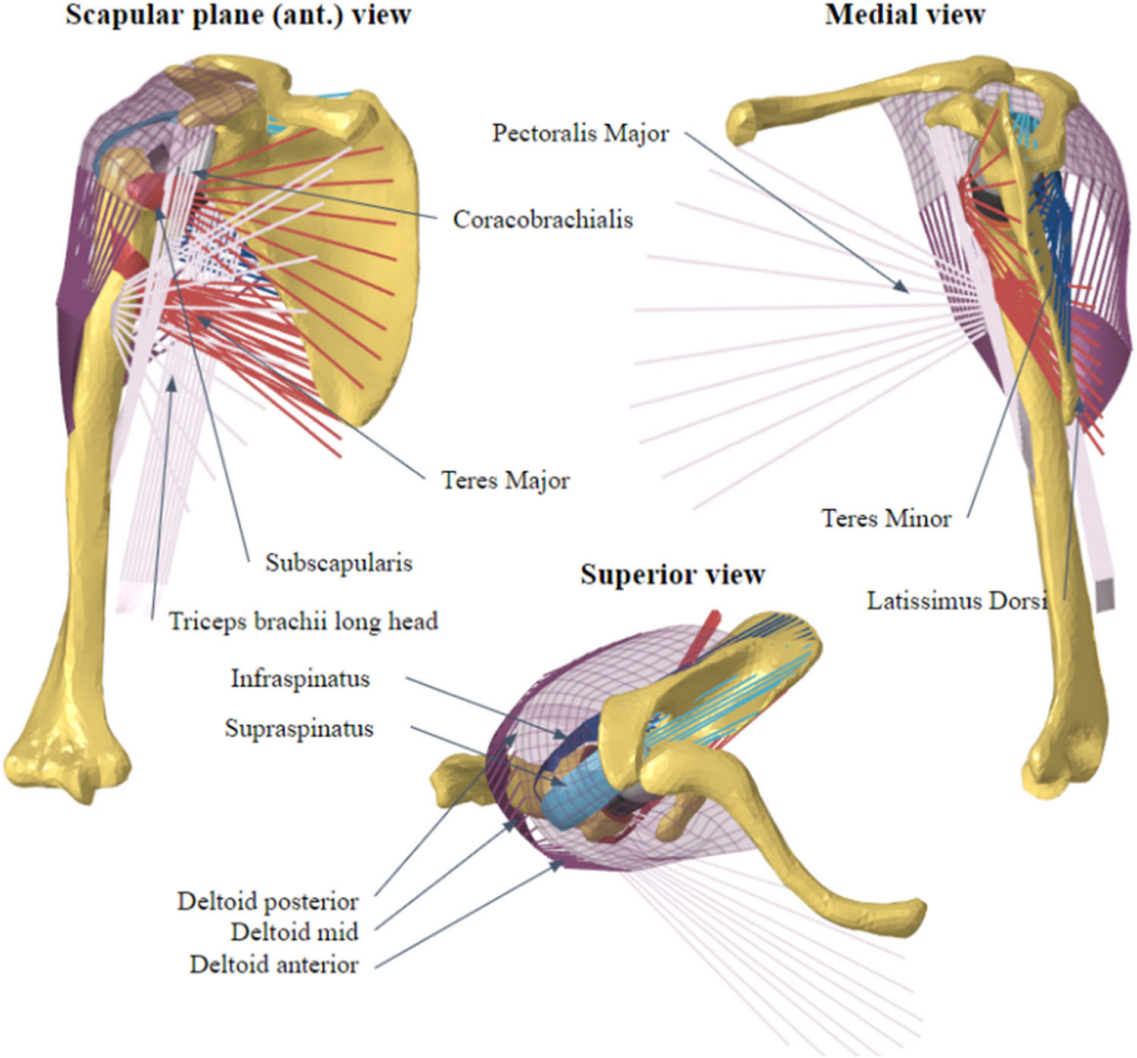
Meshed model of the humerus, scapula and clavicle including the 12 muscle bundle elements (1D active springs) and tendinous insertions (2D shell elements).

A finite element model of a glenohumeral joint was created according to the geometric measurements of a patient with no known disorders or deformities (BodyParts3D, Integrated Database Center for Life Sciences, licensed by CC Attribution‐ShareAlike 2.1). The model was morphed to match the 50th percentile bone geometry of a healthy male patient previously described by other authors [27, 36, 39]. The model included trabecular and cortical bone for the humerus, scapula and clavicle; cartilage and labrum of the glenohumeral joint; glenohumeral ligaments; and 12 muscles involved in scapulohumeral movements, including the rotator cuff and deltoid. The ligament and tendon origins and attachments were determined from the literature [14, 48] and confirmed by two independent senior surgeons (Uma Srikumaran and Geoffroy Nourissat) (Figure 1). The regional thickness of the cortical bone wall and cartilage was represented according to published morphometric measurements [10, 23, 24, 34, 38, 41, 43].

Comparisons between the model and literature references were made for scapular thickness [9], glenoid version and inclination [28, 32, 35] and glenoid cavity and humeral head curvatures [49]. These comparisons showed that the current model represented normal human morphology. The model was meshed with tetrahedral elements of 1 mm for the cartilage and labrum and 2–3 mm for the bone structures. A mesh size sensitivity analysis was performed to ensure stability of the model.

Spring 1D elements were used to model the ligaments based on descriptions of their origin and insertion areas. The resting lengths of the ligaments were defined through preliminary range of motion simulations and were found to be similar to measurements achieved by Yang et al. [48]. Tendons were modelled using triangular 2D elements. Muscles were modelled with 1D active spring elements with specific pulling forces depending on each muscle bundle (detailed hereafter). The tendons and ligaments were modelled using linear elastic mechanical properties. The bone structures were modelled using isotropic elastoplastic material laws specific to cortical and trabecular bone based on similar work by Astier et al. [4].

### Finite element model function

The simulation approach consisted of imposing muscle forces and observing glenohumeral biomechanics throughout abduction. This approach enabled us to represent the history of bone loading and to identify key stages for all observed mechanical metrics. A linear ramp of increasing force was applied in each muscle element through active stimuli to simulate abduction movement. The force ratio between each individual muscle was based on muscle cross‐sectional areas [29]. A factor was used on the forces applied in the rotator cuff muscles to reach 60 N in the rotator cuff when the middle deltoid force reached 150 N [6]. To simulate abduction, only the forces in the middle deltoid, anterior deltoid, subscapularis, supraspinatus, infraspinatus and teres minor were activated [3, 6, 19, 20, 25, 26, 33, 40, 47]. The muscle activation was increased until a scapulohumeral angle of 60° was reached, or the maximum middle deltoid force of 150 N was reached, based on muscle strength limits identified by Baumgartner et al. [6].

The distal half of the scapula and the clavicle were considered a rigid body, and their movements were fixed in all directions. The distal two‐thirds of the humerus was also considered as a rigid body. A vertical force of 25 N was applied to the distal humerus to account for upper limb weight [13]. Nonpenetration contact interfaces were defined between the humerus and the scapula, as well as between soft tissues and bones, using a point/surface penalty method with a Coulomb‐type friction coefficient of 0.2 and a minimal gap of 0.05 mm [7]. The thickness of each tendon and muscle was taken into account in these contact interfaces to provide an accurate representation of muscle moment arms [8]. The same model geometry was used to simulate two patient configurations: a healthy patient with an intact rotator cuff and a rotator cuff‐deficient patient. Deficiency was represented by deactivating forces in the supraspinatus muscle.

### Configurations and methods of measurement

**Figure 2:**
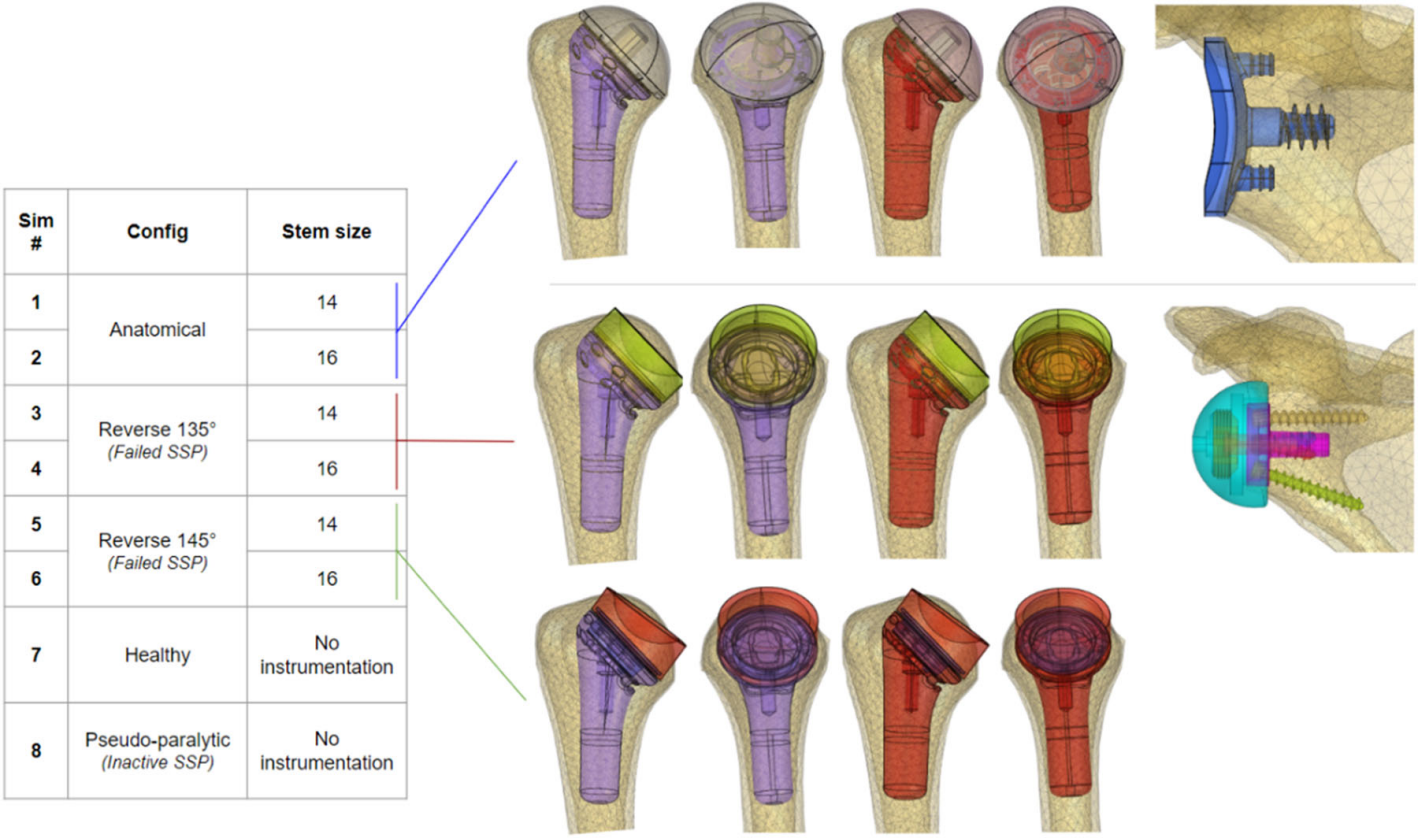
Design of experiment and description of implant placement for the total shoulder arthroplasty (TSA) configuration (top row) and reverse shoulder arthroplasty (RSA) configurations (middle and bottom row) and their corresponding glenoid counterpart.

The instrumented analysis was performed with the FX V135 (FX Shoulder Solutions) implants for two stem sizes (T14 and T16) in the following configurations (Figure 2): (1) anatomic TSA in the healthy patient and (2) RSA with symmetrical polyethylene (i.e., cylindrical with parallel faces, associated with a 135° bone cut) and asymmetrical polyethylene (i.e., cylindrical with angled faces, associated with a 145° bone cut) in the rotator cuff‐deficient patient.

Virtual surgical process included removal of ligaments, cartilage, labrum and bone resection consistent with TSA and RSA surgeries. RSA also included removal of the superior cuff. Positioning of the stem and baseplate was blindly verified by two independent senior surgeons (Uma Srikumaran and Geoffroy Nourissat) to ensure the best positioning as requested by the manufacturer. A nonpenetration contact interface was applied between the humeral and glenoid implant components, as well as between bone and implant. A total of eight simulations were performed using an explicit dynamic Finite Element Model Solver (Radioss, release 2021.1, Altair Engineering, Inc.).

For each configuration, cortical and trabecular bone von Mises stress fields in the humerus and scapula were observed and compared. Quantitative measurements were joint kinematics (scapulohumeral angle), maximal von Mises stress for bone and implant components, contact forces between individual components of the model, and glenohumeral contact force resultant vector. Finally, the volume of humeral and scapular trabecular bone where stress exceeded 0.4 MPa was also measured to provide insight into bone remodelling and stress shielding areas, which is induced by volumetric mechanical loading of trabecular bone or lack thereof, respectively. The value of 0.4 MPa was chosen to facilitate comparison between configurations.

## RESULTS

### Glenohumeral joint kinematics and kinetics

**Figure 3:**
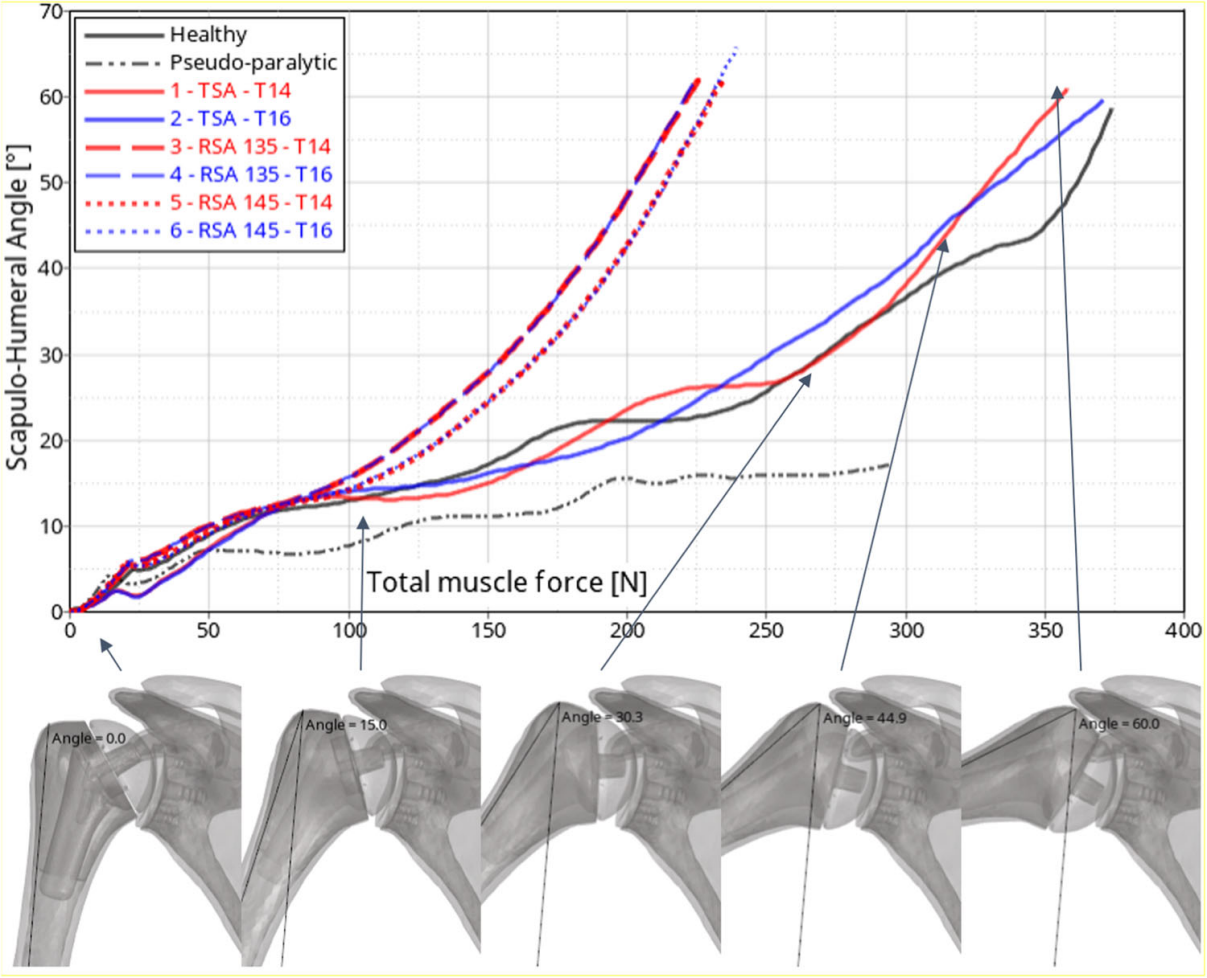
Kinematic scapulohumeral angle and force activation curve on healthy and pseudo‐paralytic model (black) and instrumented models (red and blue) and evolution of abduction motion for configuration 1—total shoulder arthroplasty (TSA)—T14 (bottom row).

The abduction kinematic analysis of the healthy and cuff‐deficient models revealed that a rupture of the supraspinatus produces a functionally limited shoulder, as expected, with only 17° of scapulohumeral angle reached at maximum muscle force (150 N in the middle deltoid). The scapulohumeral‐angle‐over‐time curves (Figure 3) of the uninstrumented models show an initial stabilization portion from 0 to 50 ms, as the coaptor muscles and weight of the arm were not given any initial state before 0 ms. Scapulohumeral angles increased with muscle activation. The healthy model reached a scapulohumeral angle of 60° (equivalent to 90° of abduction) with a total muscle force of 374 N.

The placement of both TSA and RSA (with a deficient rotator cuff) enabled full restoration of function and range of motion similar to that of a healthy shoulder. RSA in the rotator cuff‐deficient shoulder restored function regardless of stem size and polyethylene shape. After RSA, less muscle activation was required to reach 60° of scapulohumeral angle compared with TSA configurations. The symmetric polyethylene reached a 60° scapulohumeral angle with slightly lower muscle forces compared with the asymmetric polyethylene. TSA produced a glenohumeral kinematic profile similar to that of the healthy model. For each implant configuration, stem size did not affect scapulohumeral angle kinematics (Table 1).

**Table 1:** Summary of measured mechanical metrics for all simulated configurations obtained at final humeral elevation (60° glenohumeral angle).

Configuration	Head‐Glenoid contact force (*N*)	Volume of stressed trabecular bone (>0.4 MPa) (cm^3^)		Von Mises stress (MPa)						
		Humerus	Scapula	Humerus		Scapula		Implant HDPE (male)	Implant CrCo (female)	Implant other (TA6V)
				Cortical bone	Trabecular bone	Cortical bone	Trabecular bone			
1—Anat T14	311.17	20.72	3.68	37.32	7.89	18.53	3.17	7.81	163.25	228.08
2—Anat T16	287.69	11.52	4.68	18.32	7.35	19.48	3.79	7.60	92.37	410.81
3—Rev135 T14	219.22	1.50	0.42	5.47	1.61	15.75	5.24	3.11	25.69	340.12
4—Rev135 T16	215.98	1.65	0.50	5.38	2.24	16.16	5.16	2.81	25.63	479.98
5—Rev145 T14	226.15	2.78	1.28	5.99	2.68	17.03	6.23	2.58	22.77	581.38
6—Rev145 T16	218.64	3.30	0.49	6.05	3.02	16.91	4.29	2.50	20.64	308.49

*Note*: Head‐glenoid contact force relates to the total contact force between humeral and scapular implants. The implant stress data was separated by material, with HDPE corresponding to the polyethylene component, CrCo to the cobalt‐chrome component, and TA6V to all other components which are made of titanium.

**Figure 4:**
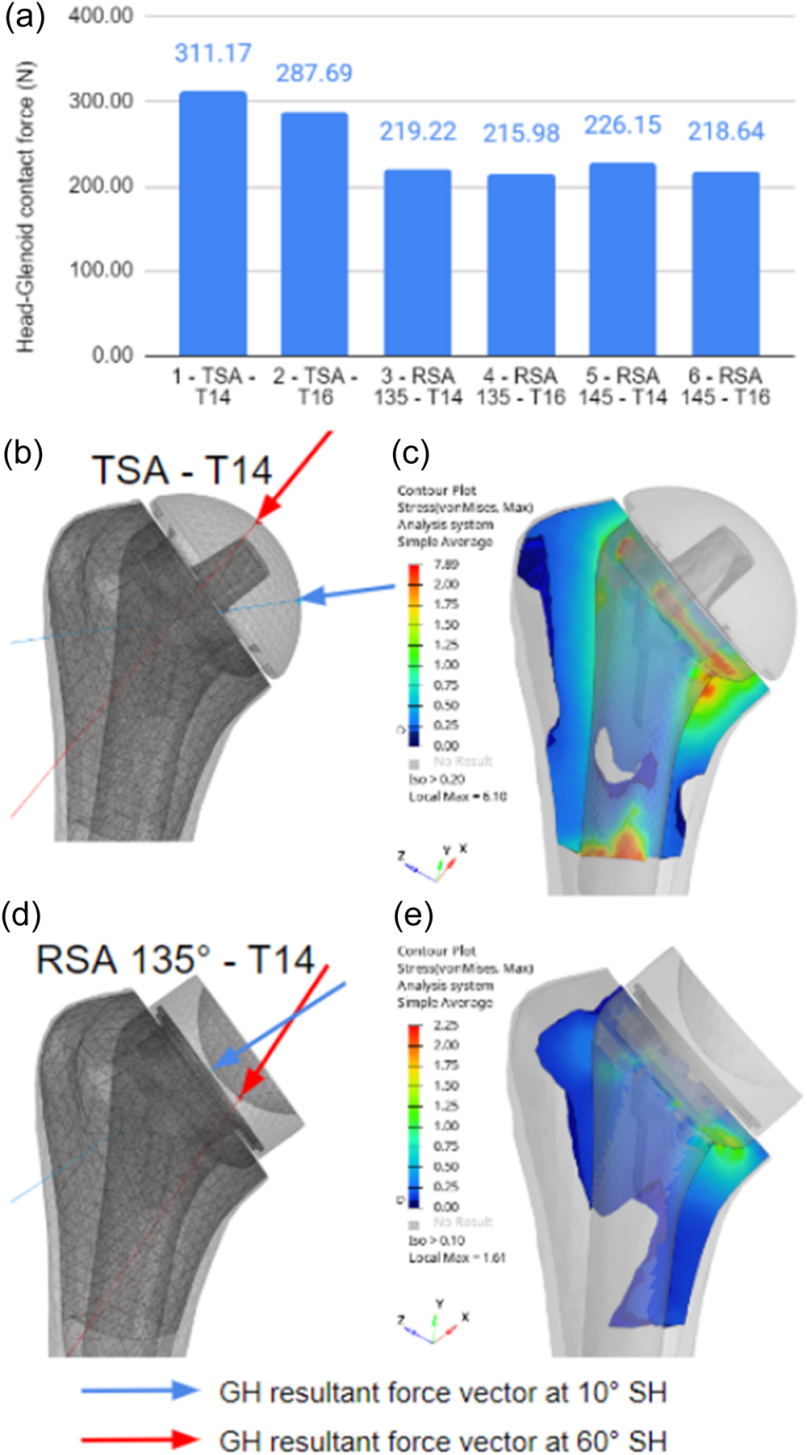
Head‐glenoid contact forces change between total shoulder arthroplasty (TSA) and reverse shoulder arthroplasty (RSA) but not in different configuration of RSA (a), glenohumeral reaction force directions at 10° and 60° abduction angle for TSA and RSA configurations (b, d), and Von Mises stress in the humeral trabecular bone superior to 0.1 MPa at 60° scapulohumeral angle (c, e).

More force was needed to reach a 60° scapulohumeral angle in the TSA model compared with the RSA model, resulting in more contact forces between the stem and the humeral trabecular bone (Figure 4a). The direction and contact point patterns were different for TSA and RSA configurations (Figure 4b–d), leading to different load distributions in the stem and adjacent bone. In the TSA configurations, the glenohumeral contact force resultant vectors were directed towards the centre of the shaft during all abduction motion. At maximum contact forces (60° scapulohumeral angle), the forces were directed towards the longitudinal axis direction of the stem. The contact forces between the trabecular bone and stem were evenly distributed around the stem shaft (Figure 4c). In RSA configurations, the glenohumeral contact force resultant vectors on the polyethylene were directed towards the centre of the stem in the early stages of abduction motion and then moved towards the medial part of the humerus, towards the calcar, as the abduction angle increased, leading to eccentric loading at maximal contact forces (60° scapulohumeral angle). The contact forces between the trabecular bone and stem were mainly located on smaller areas around the stem crown and medial to the distal tip of the stem (Figure 4e).

### Humerus stress analysis

**Figure 5:**
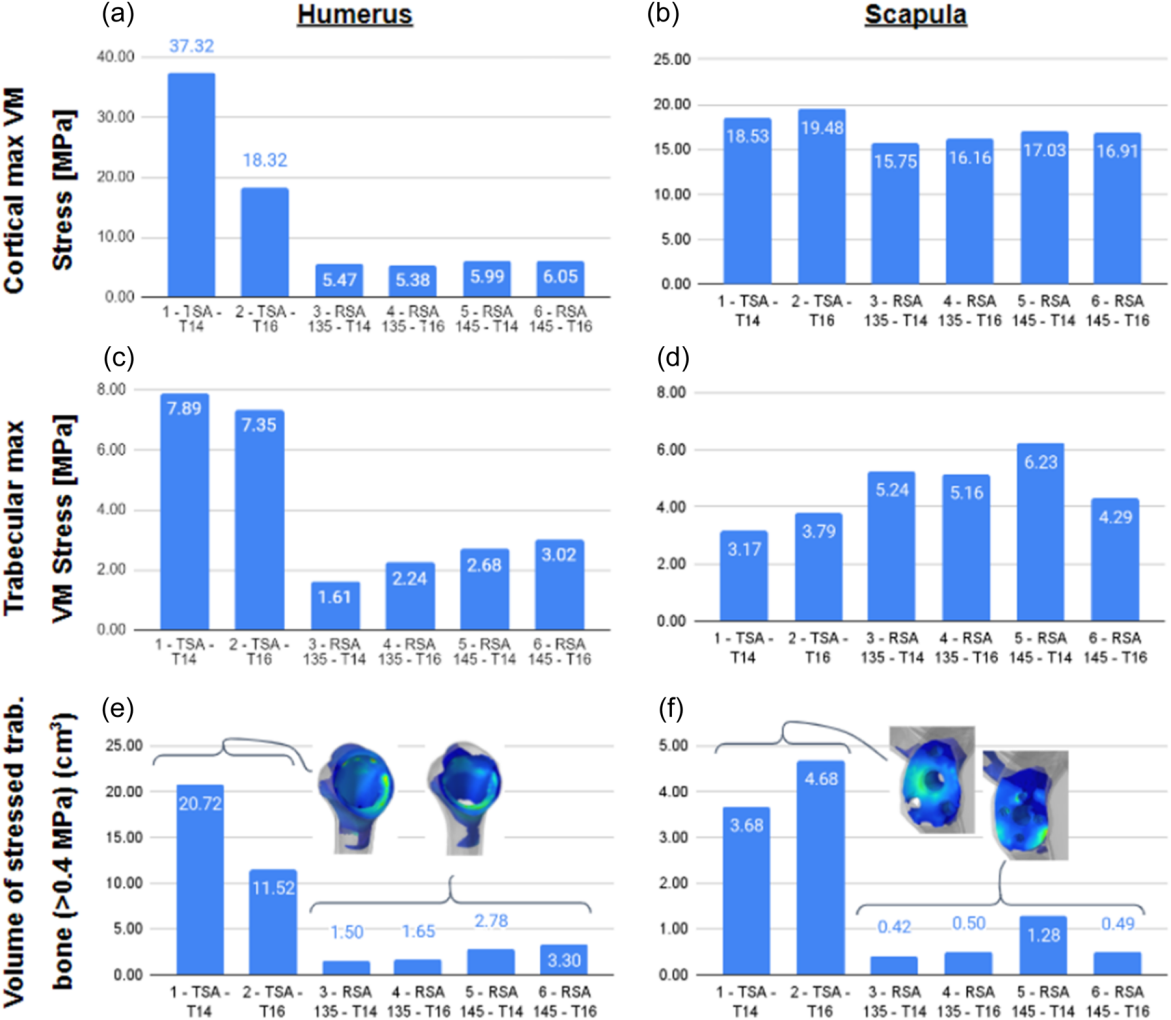
Maximal local bone stress (chart) in cortical (a, b) and trabecular (c, d) bone of scapula and humerus, and volume of trabecular bone with stress superior to 0.4 MPa (e, f).

In the TSA configurations, the higher stem size led to lower maximal local stress in the cortical and trabecular humeral bone. The stressed trabecular bone volume was also lower because there was less trabecular bone in the canal, and the forces were distributed over a larger surface area. Smaller stems led to higher stress in the proximal epiphysis close to the stem holes and metaphysis (Figure 5a,c,e).

In the RSA configurations, increasing stem size did not affect cortical bone stress but did increase the trabecular bone maximal local stress and volume of stressed bone (>0.4 MPa) of the humerus, specifically around the medial part of the stem crown. Larger stems led to higher stress in the medial proximal epiphysis. The asymmetric polyethylene led to higher trabecular bone maximal local stress and volume of stressed bone (>0.4 MPa) (Table 1).

The lowest levels of trabecular bone stress were observed around the distal half of the humeral stem. Trabecular bone stress was higher for instrumented configurations compared with preoperative models.

### Scapula stress analysis

The stress analysis on the scapula cortical bone showed slightly higher stress in TSA configurations compared with RSA because of higher muscle force activation (Figure 5a–f). There was no clear trend concerning the influence of stem size on cortical bone. In TSA configurations, the trabecular bone was less locally stressed and had a larger stressed bone volume because the polyethylene glenoid had a larger contact surface and was less rigid compared with the baseplate and screw system of the RSA configurations (Figure 5b,d,f). In RSA configurations, the high‐stress areas were located around the superior and posterior screw insertions. Polyethylene shape did not appear to influence scapular trabecular bone stress.

### Implant stress

**Figure 6:**
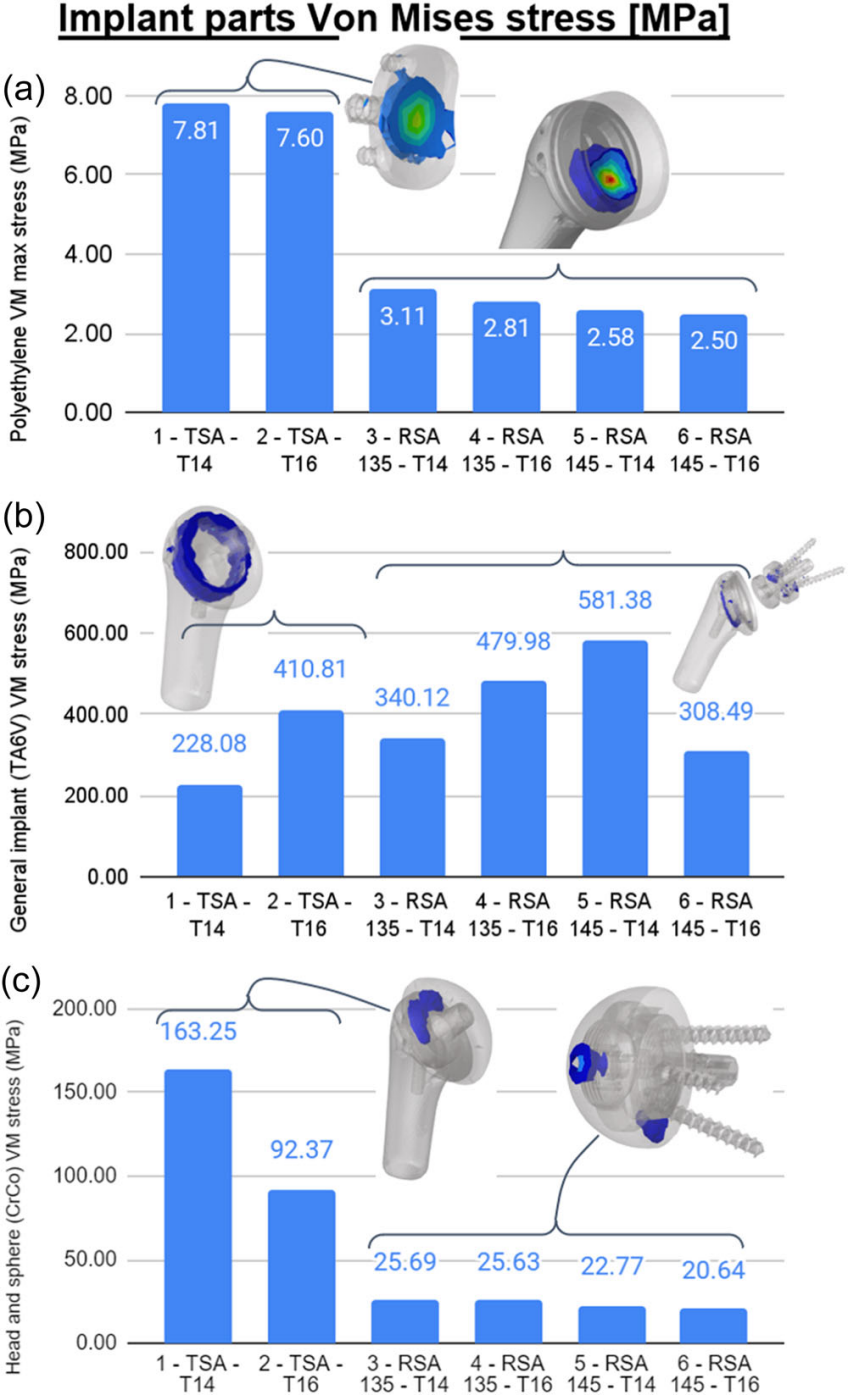
Maximal local von Mises stress in implant polyethylene (a), titanium (b) and CoCr (c) components.

Stress analysis in the implant showed that the polyethylene glenoid (in TSA) was up to three times as stressed as the polyethylene (in RSA) because the muscle activation forces were higher, and the contact area was more localized. The larger stem size (T16) showed slightly lower high local stress compared with the T14 stem. The high‐stressed areas were located at the contact with the opposed sphere/glenosphere (Figure 6a). The maximal stress level was below the yield limit of the material.

The stress analysis in the other metallic parts of the implants (titanium alloy and cobalt‐chromium alloy) showed that the high‐stressed areas were located at the connections (stem and taper or baseplate and screw connections). The maximal stress level was below the yield limit of the material (Figure 6b,c). There was no clear trend on the effect of the stem size and the metallic part maximal local stress.

## DISCUSSION

Our study demonstrates that biomechanical stress around the humeral stem differs between RSA and TSA conditions. The first important finding is that the main difference in biomechanics between TSA and RSA is caused by the glenohumeral contact force vector, which we quantified and described for the specific configuration studied here (Figure 4b,d). TSA resulted in more loading around the stem shaft, and RSA resulted in more loading around the calcar. This is an important finding suggesting that clinical survival analysis of humeral short stems must be conducted in anatomical and reverse consideration. It should be not recommended to extrapolate radiological stress shielding occurring in TSA configuration identical to RSA consideration.

The second important finding is that smaller stems seem to be more appropriate for TSA, in which stress distribution around the implant allowed a floating implant, whereas larger stems generated higher local stress concentration in the proximal lateral humeral head. Larger stems seem to be more appropriate for RSA, because most trabecular bone stress was located medially to the implant, and greater canal fill allowed a better distribution of stress throughout the bone.

An asymmetric polyethylene component induced higher stresses in the metal structures of the implant compared with a symmetric polyethylene component. Asymmetrical polyethylene resulted in a greater abduction range of motion than symmetric polyethylene components. Higher polyethylene stress in RSA compared with TSA was expected because of low congruence between the radii of glenoid and humeral components and can be a predictor of earlier material wear.

In this finite element model, a superior rotator cuff deficiency demonstrated that RSA restores function when the supraspinatus is torn and leads to lower glenohumeral reaction forces. Ackland et al. [1] used musculoskeletal models of the glenohumeral joint to evaluate the effect of rotator cuff tear severity on joint and muscle forces after RSA and to quantify the stresses at the glenoid and humeral components. They simulated abduction and flexion after RSA in different cases of intact cuff and different types of tears of the cuff. The largest calculated glenohumeral joint contact forces, muscle forces and implant stresses were higher with increasing rotator cuff deficiency, which is consistent with our results.

In the case of supraspinatus deficiency in TSA, eccentric loading and an increased humeral head translation may be observed. It has been suggested that a downward inclination of the glenoid component may balance the supraspinatus deficiency, which would be interesting to evaluate in a future study.

Although we were able to analyse the effect of stem size on bone stress distribution, the two stem sizes tested here were similar. Further evaluation of a wider range of canal fill could provide additional insight into patient‐specific osseointegration; based on bone density or humeral metaphysis individual anatomy. However, the current results are consistent with previous literature. Langohr et al. [31] performed finite element analysis to quantify the effect of varying the size of the short stem humeral components having two different diametral sizes on the changes in bone stress from the preoperative intact to the reconstructed state for loading states consistent with 45° and 75° of abduction. They found that the smaller short‐stem implant produced humeral trabecular and cortical bone stresses that were closer to the intact state. The effect of humeral implant size and canal fill on bone stresses and interface contact in the proximal humerus was also evaluated by Synnott et al. [44], who compared three generic short stems with different cross‐sectional thickness (thinner, medium and thicker) to determine the effect of stem thickness on bone contact, bone stresses and bone resorption caused by stress shielding. They found that increasing the size had no significant effects on bone‐to‐implant contact during loading, but the thinner implant with the lowest canal fill ratio produced significantly less change in stress from the intact state for both cortical and trabecular bone. The thinner implant also resulted in less stress shielding and bone resorption compared with the two other designs. Although these studies align with our results, our conclusions may not apply to all implant designs.

In a finite element analysis by Barth et al. [5], the authors analysed the influence of proximal humeral stem geometry on stress distribution and torsional stability after TSA. We found that the stem shape of the FX V135 has a large proximal base/crown, and the only shape difference is in the distal part of the stem. Thus, the stability of the stem might not change much between stem sizes.

The credibility of the finite element model used in this study was assessed by comparing several simulation outputs with phenomena and measurements reported in the literature. The glenohumeral joint forces measured were consistent with the literature [11, 37, 46], although the ranges described vary widely depending on the study or the patient. Local kinematics of the healthy preoperative model showed a superoinferior translation of the contact area of the humeral head on the glenoid, which is also consistent with the literature [12, 22, 42]. Finally, while bone stress distribution is specific to each configuration studied, we verified that our results were in the same order of magnitude compared with previous studies [12, 22]. Also, bone and implant stresses were below yield stress, which is expected because the motion is physiologic.

Despite these elements of verification and validation, our study has some limitations. Although our model enabled 3D mobility of the humerus, the configurations we studied were tested only in abduction, and the simulation of other physiological movements should be explored to provide a better understanding of implant osseointegration. Also, bone stress is not a direct indicator of bone grown or osteolysis, and further analysis should be performed to link stress distribution and levels to osseointegration, as proposed by Fernandes et al. [21].

## CONCLUSION

Our finite element study enabled quantification of differences in biomechanics between TSA and RSA. Based on the bone stress distribution analysis, we conclude that smaller stems may be more appropriate for TSA, while larger stems may be more appropriate for RSA. The current study demonstrated that different polyethylene shape on the same short stem does not increase humeral stresses but results in different ranges of motion and stresses in the metal structures of the implant.

## AUTHOR CONTRIBUTIONS

All authors developed the methodology, wrote the original draft and revised the manuscript. Geoffroy Nourissat and Uma Srikumaran supervised the study, validated the results and revised the manuscript. Léo Fradet, Rohan‐Jean Bianco and Jean‐Marie Daudet reconceptualized the study, developed the methodology, managed the project and provided resources. Léo Fradet and Rohan‐Jean Bianco performed statistical analyses, developed the methodology, programmed the software, supervised the project and wrote the original draft. Geoffroy Nourissat and Uma Srikumaran conceptualized the study, developed the methodology and revised the manuscript.

## CONFLICTS OF INTEREST STATEMENT

Geoffroy Nourissat, MD, PhD: Receiving reimbursements, fees, funding, or salary from an organization that may in any way gain or lose financially from the publication of the manuscript, either now or in the future. Victor Housset: Receiving reimbursements, fees, funding, or salary from an organization that may in any way gain or lose financially from the publication of the manuscript, either now or in the future. Jean‐Marie Daudet: Holding stocks or shares in an organization that may in any way gain or lose financially from the publication of the manuscript, either now or in the future. Léo Fradet, PhD, Eng.: Holding stocks or shares in an organization that may in any way gain or lose financially from the publication of the manuscript, either now or in the future. Rohan‐Jean Bianco, PhD.: Holding stocks or shares in an organization that may in any way gain or lose financially from the publication of the manuscript, either now or in the future. Uma Srikumaran, MD: Receiving reimbursements, fees, funding, or salary from an organization that may in any way gain or lose financially from the publication of the manuscript, either now or in the future.

## ETHICS STATEMENT

Not applicable.

## Data Availability

Due to commercial restrictions, supporting data are not available. The data sets used and/or analysed during the current study are available from the corresponding author upon reasonable request.
